# Comparison of pregnancy outcomes between women with early‐onset and late‐onset gestational diabetes in a retrospective multi‐institutional study in Japan

**DOI:** 10.1111/jdi.13101

**Published:** 2019-06-27

**Authors:** Tomoka Usami, Maki Yokoyama, Megumi Ueno, Noriyuki Iwama, Norimasa Sagawa, Reo Kawano, Masako Waguri, Hiroshi Sameshima, Yuji Hiramatsu, Takashi Sugiyama, for the Japan Diabetes and Pregnancy Study Group

**Affiliations:** ^1^ Department of Obstetrics and Gynecology Ehime University Graduate School of Medicine Toon Japan; ^2^ Center for Perinatal Medicine Tohoku University Hospital Sendai Japan; ^3^ Department of Obstetrics and Gynecology Rakuwakai Otowa Hospital Kyoto Japan; ^4^ Center for Integrated Medical Research Hiroshima University Hospital Hiroshima Japan; ^5^ Department of Maternal Internal Medicine Osaka Medical Center and Research Institute for Maternal and Children Health Osaka Japan; ^6^ Department of Obstetrics and Gynecology Miyazaki University School of Medicine Miyazaki Japan; ^7^ Department of Obstetrics and Gynecology Okayama City General Medical Center Okayama Japan

**Keywords:** Gestational diabetes mellitus, Management, Pregnancy outcome

## Abstract

**Aims/Introduction:**

To compare pregnancy outcomes between women with gestational diabetes mellitus (GDM) diagnosed early and late in pregnancy in Japan.

**Materials and Methods:**

We examined women diagnosed with GDM in this multi‐institutional retrospective study. Women were divided into two groups by gestational age at diagnosis: <24 weeks of gestation (early group, 14.4 ± 4.2 weeks) and ≥24 weeks of gestation (late group, 29.6 ± 3.4 weeks). Dietary counseling with self‐monitoring of blood glucose with or without insulin therapy was initiated for both groups. Pregnancy outcomes were compared between the groups.

**Results:**

Data from 600 early and 881 late group participants from 40 institutions were included. Although pre‐pregnancy body mass index was higher in the early group than in the late group, gestational weight gain was lower in the early group. Hypertensive disorders of pregnancy and cesarean section were more prevalent in the early than in the late group (9.3% vs 4.8%, *P* < 0.001; 34.2% vs 32.0%, *P* < 0.001, respectively). The prevalence of large‐for‐gestational‐age infants was higher in the late than in the early group (24.6% vs 19.7%, respectively, *P* = 0.025). There was no significant difference in other neonatal adverse outcomes between the groups. Multiple logistic regression analysis showed that early group, nulliparity and pre‐pregnancy body mass index were associated with hypertensive disorders of pregnancy.

**Conclusions:**

These results suggest that maternal complications, including hypertensive disorders of pregnancy and cesarean delivery, were higher in the early group than in the late group. Earlier intervention for GDM might be associated with a reduction in large‐for‐gestational‐age infants.

## Graphical abstract

The prevalence of hypertensive disorders of pregnancy was higher in the early group than in the late group. Similarly, the prevalence of cesarean section was higher in the early group than in the late group. The prevalence of large‐for‐gestational‐age infants was higher in the late group than in the early group.

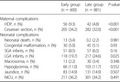

## Introduction

Gestational diabetes mellitus (GDM) is known as glucose intolerance that first develops or is found during pregnancy; however, the gestational age at diagnosis of GDM is not always reported^1^. Recently, the prevalence of obesity and type 2 diabetes mellitus among childbearing women has increased significantly, because some women are diagnosed with pre‐existing diabetes at their first visit to a hospital or clinic. These changes have led the American Diabetes Association to redefine GDM to include only glucose intolerance diagnosed in the second or third trimester of gestation. This definition excludes women with pre‐existing diabetes or those diagnosed before the second trimester^2^.

Gestational diabetes mellitus can lead to maternal complications, such as hypertensive disorders of pregnancy (HDP) and cesarean delivery, as well as infant complications, such as large‐for‐gestational‐age (LGA) infants, macrosomia, respiratory distress syndrome, neonatal hypoglycemia and neonatal jaundice^3^, ^4^. LGA infants often experience perinatal complications of GDM^5^, ^6^, and the mean glucose concentration of a mother with GDM has a strong impact on birthweight^7^. The Hyperglycemia Adverse Pregnancy Outcome study has shown that the level of maternal hyperglycemia is correlated with adverse maternal and perinatal outcomes^3^. On the basis of the Hyperglycemia Adverse Pregnancy Outcome study, new criteria for the GDM were proposed by the International Association of Diabetes in Pregnancy Study Group (IADPSG) in 2010^8^. These criteria, based primarily on plasma glucose levels associated with a 1.75‐fold increase in the risk of adverse pregnancy outcomes, such as delivery of LGA infants and cesarean delivery^8^, include initial diagnosis of women with GDM before 24 weeks of gestation. The goal of an initial test in early pregnancy is to identify “overt diabetes in pregnancy,” also known as “undiagnosed pre‐existing diabetes in early pregnancy^8^,” because overt diabetes in pregnancy is known to be associated with adverse complications compared with GDM^9^. In contrast, the appropriate diagnostic criteria for the diagnosis of GDM in early pregnancy have not been clarified, and the effect of early diagnosis and intervention on the risk of adverse pregnancy outcomes has also not been shown.

In Japan, GDM has been screened for and diagnosed early in pregnancy for >20 years. However, it is not clear whether pregnancy outcomes can be improved by managing women with GDM early in pregnancy versus screening them for GDM in late pregnancy. In fact, there are no reports that compare adverse pregnancy outcomes between women with GDM diagnosed in the first half of pregnancy and second half of pregnancy. Therefore, we compared pregnancy outcomes of women with GDM diagnosed at <24 weeks of gestation and those diagnosed at ≥24 weeks of gestation using the Japan Diabetes and Pregnancy Study Group database.

## Methods

### Participants and study design

We carried out a multicenter retrospective study using databases from 40 institutions in Japan over the period from 2003 to 2009. All participants provided written informed consent. The ethics committee at each of the collaborating centers of the Japan Diabetes and Pregnancy Study approved the study design and protocol. Other studies based on clinical questions have been published previously using the same database as that used in the Japan Diabetes and Pregnancy Study^9^, ^10^, ^11^. In the present study, women with GDM were divided into two groups by gestational age at diagnosis: <24 weeks of gestation (early group) and ≥24 weeks of gestation (late group).

Singleton pregnant women with no history of GDM were included. Women with multi‐fetal gestations, pre‐gestational diabetes including type 1 or type 2 diabetes mellitus, GDM treated previously or active chronic systemic disease including hyperthyroidism, systemic lupus erythematosus, and rheumatoid arthritis were excluded. Women with overt diabetes in pregnancy, as defined by IADPSG, were excluded. Overt diabetes in pregnancy was defined as two abnormal values on the 75‐g oral glucose tolerance test (OGTT); fasting glucose level 126 mg/dL and 2‐h postprandial glucose level 200 mg/dL; glycated hemoglobin level 6.5%; random glucose level 200 mg/dL; or diabetic retinopathy recognized in pregnancy. In addition, patients with fulminant type 1 diabetes mellitus were excluded. All pregnant women participating in the present study underwent a universal two‐step screening process for diagnosis of GDM both in early and late pregnancy. This test included a random glucose test in early pregnancy and a random glucose test or a 50‐g glucose challenge test between 24 and 32 weeks of gestation, respectively. Those with a random glucose test result ≥100 mg/dL or glucose challenge test result ≥140 mg/dL required a diagnostic test for GDM; that is, 75‐g OGTT. On the basis of the former Japan Society of Obstetrics and Gynecology (JSOG) criteria, women meeting more than two of the following OGTT cut‐off points were considered to have GDM: fasting 100 mg/dL; 1 h 180 mg/dL; and 2 h 150 mg/dL^12^.

The database of clinical background characteristics included parity, maternal age, pre‐pregnancy body mass index (BMI), gestational weight gain, gestational age at delivery, delivery mode including vaginal delivery or cesarean section, and infant parameters including infant sex, birth weight, Apgar score (1 and 5 min after birth), perinatal mortality and congenital malformations. In terms of maternal pregnancy complications, HDP, including pre‐eclampsia, gestational hypertension and chronic hypertension, were examined. Pre‐pregnancy bodyweight was determined based on self‐reporting at the first prenatal visit. Gestational age was determined based on the last menstrual period or measurement of crown–rump length using ultrasound in early pregnancy. Gestational hypertension was defined as sustained blood pressure readings of ≥140/90 mmHg during pregnancy after 20 weeks of gestation after a previously normotensive status without the feature of pre‐eclampsia, which normalized by 12 weeks postpartum. Pre‐eclampsia was defined as a condition of hypertension accompanied by at least one of the complications as shown, following new onset after 20 weeks of gestation, with all symptoms normalizing by 12 weeks postpartum: proteinuria; other maternal organ dysfunctions, such as liver involvement without any underlying chronic diseases, progressive kidney dysfunction, stroke and neurological complications; hematological complications; and uteroplacental dysfunction. Chronic hypertension was defined as a condition of hypertension diagnosed before pregnancy or before 20 weeks of gestation without features of superimposed pre‐eclampsia. Macrosomia was defined as a birthweight of ≥4,000 g. LGA infants were defined as those with a birthweight within or above the 90th percentile of the birthweight of Japanese infants^13^. Small‐for‐gestational‐age infants were defined as those with a birthweight less than the 10th percentile of the birthweight of Japanese infants^13^. Congenital malformations were defined as having a morphological abnormality with functional impairment. For instance, congenital malformations included congenital heart diseases, such as atrial septal defect and ventricular septal defect, neural tube defects including spina bifida with or without meningocele, and atresia of the upper digestive tract.

Women with GDM received guidance regarding self‐monitoring of blood glucose levels three to six times a day from a licensed nurse. Dietary counseling was provided for each woman with GDM. Briefly, a registered dietician examined the daily dietary intake of women with GDM using the recollection method and instructed women on the appropriate gestational weight gain on the basis of their pre‐pregnancy BMI. The JSOG recommends an additional 200 kcal per day for non‐obese women during pregnancy in addition to 30 kcal/kg of non‐pregnant ideal bodyweight^14^. No additional caloric intake was prescribed during pregnancy for overweight and obese women with GDM. Ideal bodyweight was defined by the data of the Japan Ministry of Health, Labor and Welfare^15^. On the basis of self‐monitoring of blood glucose, insulin therapy was started if fasting glucose levels <95 mg/dL and 2‐h postprandial levels <120 mg/dL were not achieved.

### Study outcomes

Maternal adverse outcomes included HDP comprising gestational hypertension, pre‐eclampsia, chronic hypertension and cesarean section. Neonatal adverse outcomes included neonatal death and complications associated with maternal hyperglycemia, such as delivery of LGA infants, macrosomia, infant hypoglycemia, infant jaundice, respiratory distress syndrome and admission to the neonatal intensive care unit. Blood sampling for neonatal glucose measurement was collected 1 or 2 h after birth. Neonatal hypoglycemia was defined as blood glucose levels <35 mg/dL. Neonatal hyperbilirubinemia was defined as the requirement of phototherapy.

### Statistical analysis

Baseline clinical characteristics and measurements of biomarkers in both the early GDM group and the late GDM group are presented as the mean ± standard deviation and as either medians or percentages. Univariate tests to assess differences between groups were carried out using the χ^2^‐test. Also, variables with a significant difference between any two groups were then included in multiple logistic regression analysis. *P*‐values <0.05 (two‐tailed) were considered statistically significant. Statistical analyses were carried out using SAS version 9.4 (SAS Institute, Cary, NC, USA).

## Results

A total of 1,806 women in 40 institutions were diagnosed with GDM from 2003 through 2009. Of the 1,806 pregnancies, 325 were excluded because of inadequate data, multiple pregnancies or chromosomal abnormalities in infants. In total, 1,481 women with GDM were included in the study. These women were divided into two groups on the basis of gestational age at GDM diagnosis: the early group (<24 weeks; *n* = 600) and the late group (≥24 weeks, *n* = 881).

Table 1 shows the baseline characteristics of the women. Maternal age in the early GDM group was older than in the late GDM group (34.0 ± 4.9 years vs 33.4 ± 4.8 years, *P* = 0.032). The prevalence of nullipara showed no significant differences between groups. Pre‐pregnancy BMI was significantly higher in the early group than in the late group (26.2 ± 6.2 vs 24.1 ± 5.2, *P* < 0.001). Gestational weight gain was lower in the early group than in the late group (4.9 ± 5.8 kg vs 7.3 ± 4.8 kg, *P* = 0.032). Gestational age at diagnosis of GDM was different between groups (14.4 ± 4.2 weeks vs 29.6 ± 3.4 weeks, *P* < 0.001). Although gestational age showed no significant difference between groups, birthweight was heavier in the late group than in the early group (2928.2 ± 641.2 g vs 3012.2 ± 640.8 g, *P* = 0.013). Both fasting plasma glucose levels and plasma glucose levels at 1 h after 75‐g OGTT were higher in the early group than in the late group (202.4 ± 30.2 mg/dL vs 198.4 ± 26.7 mg/dL, respectively, *P* = 0.005).

**Table 1:** Clinical characteristics

	Early group (*n* = 600)	Late group (*n* = 881)	*P*‐value
Maternal age (years)	34.0 ± 4.9	33.4 ± 4.8	0.032
Nullipara, *n* (%)	421 (47.8%)	459 (52.2%)	0.672
Pre‐pregnancy BMI	26.2 ± 6.2	24.1 ± 5.2	<0.001
Gestational weight gain (kg)	4.9 ± 5.8	7.3 ± 4.8	<0.001
Gestational age at diagnosis (weeks)	14.4 ± 4.2	29.6 ± 3.4	<0.001
Gestational weeks at delivery (weeks)	38.0 ± 2.2	38.1 ± 2.0	0.267
Birthweight (g)	2,928.2 ± 641.2	3,012.2 ± 640.8	0.013
Results of 75 g OGTT (mg/dL)			
Fasting PG	92.2 ± 12.0	90.8 ± 12.8	0.035
1‐h PG	202.4 ± 30.2	198.4 ± 26.7	0.005
2‐h PG	175.0 ± 29.4	172.6 ± 26.7	0.104

Data are expressed as mean (standard deviation) or percentages unless otherwise noted. Large‐for‐gestational‐age was defined as a birthweight greater than the 90th percentile for Japanese infants. BMI, body mass index; OGTT, oral glucose tolerance test; PG, plasma glucose.

Maternal and neonatal complications are shown in Table 2. The prevalence of HDP (9.3% vs 4.8%, *P* < 0.001) and cesarean section (34.2% vs 32.0%, *P* < 0.001) were higher in the early group than in the late group. The prevalence of small‐for‐gestational‐age infants was not significantly different between groups. The prevalence of LGA infants was higher in the late group than in the early group (19.7% vs 24.6%, *P* = 0.025). Other neonatal complications, such as neonatal death, congenital malformation, macrosomia, neonatal hypoglycemia, neonatal jaundice and admission to the neonatal intensive care unit, showed no significant difference between the two groups.

**Table 2:** Maternal and neonatal complications

	Early group (*n *= 600)	Late group (*n* = 881)	*P*‐value
Maternal complications			
HDP, *n* (%)	56 (9.3)	42 (4.8)	<0.001
Cesarean section, *n* (%)	205 (34.2)	282 (32.0)	<0.001
Neonatal complications			
Neonatal death, *n* (%)	13 (3.4)	19 (2.2)	0.981
Congenital malformation, *n* (%)	30 (5.0)	45 (5.1)	0.93
SGA infants, *n* (%)	51 (8.5)	57 (6.5)	0.16
LGA infants, *n* (%)	118 (19.7)	217 (24.6)	0.025
Macrosomia, *n* (%)	13 (2.2)	30 (3.4)	0.348
Hypoglycemia, *n* (%)	66 (11.0)	103 (11.7)	0.552
Jaundice, *n* (%)	83 (14.9)	131 (14.9)	0.507
NICU, *n* (%)	217 (36.2)	301 (34.2)	0.491

Large‐for‐gestational‐age (LGA) was defined as a birthweight greater than the 90th percentile for Japanese infants. HDP, hypertensive disorders of pregnancy; NICU; neonatal intensive care unit; SGA, small‐for‐gestational‐age.

Table 3 shows the risk factors for HDP based on a cohort identified as having GDM identified by multiple logistic regression analysis. Maternal age at delivery, nulliparity, early group, pre‐gestational BMI, gestational weight gain and plasma glucose levels at 2 h after 75‐g OGTT were associated with the onset of HDP.

**Table 3:** Risk factors for hypertensive disorders of pregnancy

	β	SE (β)	*P*	OR	95% CI
Maternal age	0.058	0.022	0.009	1.060	1.015–1.107
Nullipara	0.661	0.226	0.003	1.937	1.245–3.014
Early group	0.639	0.227	0.005	1.895	1.215–2.956
Pre‐pregnancy BMI	0.128	0.018	<0.0001	1.137	1.097–1.179
Gestational weight gain	0.099	0.020	<0.0001	1.105	1.061–1.150
1‐h PG of OGTT	−0.002	0.0042	0.698	0.998	0.990–1.007
2‐h PG of OGTT	0.013	0.0040	0.001	1.013	1.005–1.021

Area under the curve 0.761. BMI, body mass index; CI, confidence interval; OGTT, oral glucose tolerance test; OR, odds ratio; PG, plasma glucose; SE, standard error.

Table 4 shows the associated factors for LGA infants based on a cohort identified as having GDM by multiple logistic regression analysis. Maternal age at delivery was found to be associated with LGA infants. In contrast, the GDM group, pre‐pregnancy BMI, gestational weight gain and plasma glucose levels at 2 h after 75‐g OGTT were not associated with LGA infants.

**Table 4:** Risk factors for large‐for‐gestational‐age infant

	β	SE (β)	*P*	OR	95% CI
Maternal age	0.030	0.013	0.023	1.031	1.004–1.058
Late group	0.215	0.134	0.110	1.240	0.953–1.613
Pre‐pregnancy BMI	−0.014	0.012	0.276	0.987	0.963–1.011
Gestational weight gain	0.010	0.013	0.469	1.010	0.984–1.036
2‐h PG of OGTT	−0.003	0.002	0.227	0.997	0.993–1.002

Area under the curve 0.563. BMI, body mass index; CI, confidence interval; OGTT, oral glucose tolerance test; OR, odds ratio; PG, plasma glucose; SE, standard error.

## Discussion

The present study showed that women with GDM diagnosed in early pregnancy had a higher prevalence of maternal complications, including HDP and cesarean section, whereas women with GDM diagnosed in late pregnancy had a higher prevalence of LGA infants. These findings might be due to the possibility that earlier initiation of treatment results in a reduction in LGA infants.

The results of the present study are partially in agreement with those of previous reports. For instance, Sweeting *et al*.^16^ reported that despite early diagnostic OGTT and current treatment for GDM, women with GDM diagnosed before 24 weeks of gestation had more adverse pregnancy outcomes, including a higher prevalence of HDP, preterm delivery, cesarean section and neonatal jaundice compared with women with GDM diagnosed after 24 weeks of gestation. However, in their study, women who underwent an OGTT early in pregnancy also had multiple risk factors for GDM. Thus, women diagnosed with GDM before 24 weeks of gestation were at high risk for GDM, unlike participants in the present study who underwent routine screening for GDM.

There is no clear evidence of a reduction in the prevalence of poor pregnancy outcomes with early treatment in women with GDM diagnosed early in pregnancy. In a subanalysis of a multi‐institutional randomized trial for a mild degree of GDM^17^, Palatnik *et al*.^18^ reported that earlier intervention for mild GDM was not related to pregnancy outcomes compared with non‐intervention. However, in their study, treatment was initiated from 24 weeks of gestation. Therefore, we cannot know the effects of treatment for women with GDM diagnosed before 24 weeks of gestation.

In the present study, the frequency of LGA infants was higher in the late group than in the early group. Notably, duration of treatment in women in the early group was longer than that in the late group. As a result, gestational weight gain in the early group was lower than that in the late group, leading to a higher frequency of LGA in the late group. In addition, although the prevalence of HDP was higher in the early group than the late group, there was no significant difference in the prevalence of LGA infants between the two groups. Therefore, the reason the frequency of LGA infants is lower in the early group than in the late group would not be related to HDP causing small‐for‐gestational‐age infants. The prevalence of LGA infants in the early group was still high (19.7%) compared with the general population. This might have resulted from the effect of maternal BMI, because maternal BMI is known to be independently associated with birthweight and delivery of LGA infants^4^, ^19^. In contrast, in the late group, the gestational weeks at diagnosis was almost 30 weeks of gestation. In this case, most of the women with GDM diagnosed in the first half of pregnancy received treatment from 31 or 32 weeks of gestation, suggesting that good glycemic control could not be achieved at 32 weeks of gestation. Lin *et al*.^20^ and Sameshima *et al*.^21^ showed that if women with diabetes achieve good glycemic control before 32 weeks of gestation, the number of LGA infants can be reduced^20^, ^21^. In the present study, there is a possibility that good glycemic control was not achieved until delivery in the late group. Therefore, earlier intervention in the first half of gestation could reduce the prevalence of LGA infants.

The pathophysiological aspect of GDM is also important. It has been reported that both early and late GDM were associated with impairments in β‐cell function^22^, ^23^. Obesity can lead to insulin resistance, which might result in early GDM^24^. A similar pathophysiological condition might have existed in the current study, as the early group included women with higher BMI compared with the late group. Being overweight (25 ≤ pre‐gestational BMI < 30) or obese (pre‐gestational BMI ≥30) has also been known to have additional negative outcomes on pregnancy, such as HDP and cesarean section, as well as perinatal complications, such as delivery of LGA infants^25^. In the present study, the early group included women with higher BMI, perhaps leading to a higher prevalence of HDP. In fact, the early group and pre‐pregnancy BMI were risk factors for HDP in the present study. Therefore, a screening test for GDM early in pregnancy might be an effective tool to identify the high‐risk group for HDP. The reason maternal age was associated with LGA infants is unclear. Past reports have not shown a positive association between maternal age and LGA infants in women with GDM. In contrast, pre‐gestational BMI and gestational weight gain, which are important risk factors for LGA infants, were not associated with delivery of LGA infants in this study, suggesting that glycemic control at the time of delivery might be different between the two groups. This is one of the limitations of the present study.

Maternal and perinatal adverse outcomes are directly associated with the degree of hyperglycemia^26^. In this regard, the degree of glucose intolerance was different between women who met the former JSOG criteria for GDM in the present study compared with that in those who met the IADPSG criteria. Women with GDM who meet the IADPSG criteria could have a milder form of GDM compared with women who meet the JSOG criteria used in the present study. In fact, Hagiwara *et al*.^27^ reported that treatments started after GDM diagnosis in early pregnancy based on IADPSG criteria showed no effectiveness compared with treatments initiated after GDM diagnosis in the latter half of gestation. However, they did not compare the therapeutic method between early in pregnancy and late in pregnancy in the early‐onset GDM. Therefore, another limitation of this retrospective study is that their results cannot provide the effectiveness of treatment for the early group. In contrast, Alunni *et al*.^28^ showed that women with GDM diagnosed early in pregnancy on the basis of the IADPSG criteria require pharmacotherapy more frequently than those diagnosed later in pregnancy, implying a more severe form of hyperglycemia. These results are not in agreement. The present results also cannot provide evidence of usefulness of early intervention for the early GDM group. Therefore, prospective, randomized, controlled trials are necessary to delineate which women require intervention in early pregnancy.

Some limitations must be considered in interpreting the data in the present study. First, we could not compare pregnancy outcomes between women with GDM and women with normal glucose tolerance, because we only included women with GDM. Second, we could not ascertain whether glycemic control for GDM in the two groups was appropriate or similar at the time of delivery. Therefore, we could not assess the exact effect of glycemic control on the incidence of LGA infants. Also, as the participants were recruited on the basis of the former JSOG criteria for GDM, we did not compare real pregnancy outcomes between women with GDM based on the previous JSOG criteria and women with GDM based on the IADPSG criteria.

In summary, in the present study, maternal complications, such as HDP and cesarean section, were associated with early GDM. The use of self‐monitoring of blood glucose, dietary counseling and insulin therapy could possibly reduce the incidence of LGA infants. A reduction in the number of LGA infants is particularly important, because LGA not only places the infants at a high risk for perinatal complications, but also might contribute to metabolic syndrome later in life. Further studies focusing on weight control before conception, dietary counseling in terms of specific nutrients and recommendations regarding diet for LGA infants are required. In addition, randomized controlled trials on the treatment of early‐onset GDM are required to ascertain the effectiveness of such treatment.

## Disclosure

The authors declare no conflict of interest.

## Appendix

The contributors of the Japan Diabetes and Pregnancy Study Group include Hirosaki University Graduate School of Medicine; Nishisaitama‐Chuo National Hospital; Asahi General Hospital; NTT East Hospital; Keio University School of Medicine; Tokyo Medical and Dental University; Tokyo Women's University School of Medicine; Tokyo Medical School of Medicine Hachioji Medical Center; National Center for Child Health and Development; Saiseikai Yokohamashi Tobu Hospital; St. Marianna University School of Medicine; Yokohama City University Medical Center; Toyama University Graduate School of Medicine; Shinshu University Graduate School of Medicine; Fukui University Graduate School of Medicine; Fukui Prefectural Hospital; Mie University Graduate School of Medicine; Ise Red Cross Hospital; Shiga University of Medical Science; Kyoto University School of Medicine, Kyoto Prefectural University of Medicine; Osaka University School of Medicine; Osaka Medical Center and Research Institute for Maternal and Children Health; Nara Medical University; Kobe University School of Medicine; Hyogo Prefectural Kobe Children's Hospital; Himeji Red Cross Hospital; Okayama Medical Center; Hiroshima University Graduate School of Medicine; Tottori University Graduate School of Medicine; Ehime University School of Medicine; Ehime Prefectural Central Hospital; Kurume University School of Medicine; Oita University Graduate School of Medicine; Nagasaki University Graduate School of Medicine; National Hospital Organization Nagasaki Medical Center; Nagasaki City Hospital Organization Nagasaki Municipal Hospital; Kumamoto City Hospital; Miyazaki University Graduate School of Medicine; and Okinawa Chubu Hospital.
